# Abnormal structural brain network and hemisphere-specific changes in bulimia nervosa

**DOI:** 10.1038/s41398-019-0543-1

**Published:** 2019-08-27

**Authors:** Li Wang, Kun Bi, Jing An, Meng Li, Ke Li, Qing-Mei Kong, Xue-Ni Li, Qing Lu, Tian-Mei Si

**Affiliations:** 1 Department of Neurology, Xuanwu Hospital, Capital Medical University, Beijing, China; 2 Peking University the Sixth Hospital, Institute of Mental Health; National Clinical Research Center for Mental Health Disorders & Key Laboratory of Mental Health, Ministry of Health, Peking University, Beijing, China; 3 Key Laboratory of Child Development and Learning Science, School of Biological Sciences & Medical Engineering, Southeast University, Nanjing, China; 4 Beijing Huilongguan Hospital, Beijing, China; 5 Zhengzhou Eighth People’s Hospital, Zhengzhou Mental Health Center, Zhengzhou, Henan China; 6 Department of Radiology, 306 Hospital of People’s Liberation Army, Beijing, China

**Keywords:** Psychiatric disorders, Neuroscience

## Abstract

Bulimia nervosa (BN) is characterized by episodic binge eating and purging behaviors. Disrupted neural processes of self-regulation, taste-rewarding, and body image has been associated with the pathogenesis of BN. However, the structural basis for these behavioral and functional deficits remains largely unknown. We employed diffusion tensor imaging and graph theory approaches (including the nodal properties and network-based statistics (NBS)) to characterize the whole-brain structural network of 48 BN and 44 healthy women. For nodal measures of strength, local efficiency, and betweenness centrality, BN patients displayed abnormal increases in multiple left-lateralized nodes within the mesocorticolimbic reward circuitry (including the orbitofrontal cortex, anterior cingulate, insular, medial temporal, and subcortical areas), lateral temporal-occipital cortex, and precuneus, while reduced global efficiency was observed in the right-lateralized nodes within the dorsolateral prefrontal cortex, mesocorticolimbic circuitry, somatosensory and visuospatial system. Several mesocorticolimbic nodes significantly correlated with BN symptoms. At a network level, we found increased left-lateralized connections primarily within the orbitofrontal cortex and its connections to mesocorticolimbic and lateral temporal-occipital areas, but reduced right-lateralized connections across the inferior frontal gyrus and insula, as well as their connections to the lateral temporal cortex. This study revealed BN-related changes in white-matter connections across the prefrontal control, mesocorticolimbic reward, somatosensory and visuospatial systems. The hemispheric-specific change could be an important aspect of the pathophysiology of BN. By characterizing whole-brain structural network changes of BN, our study provides novel evidence for understanding the behavioral and functional deficits of the disorder.

## Introduction

Bulimia nervosa (BN) is a severe psychiatric syndrome characterized by episodic binge eating and purging behaviors. Existing treatments—both cognitive behavior therapy and pharmacotherapy, have at most 30–40% efficient rate for BN^1^. The unclear neurobiological underpinnings have largely hindered the development of more effective treatments for the disorder.

Difficulties in several psychopathological dimensions, including self-regulation or impulsive control, taste-rewarding, and body image processing, were considered core factors that promote the initiation and maintenance of binge eating and purging behaviors^2^. Studies using functional MRI (fMRI) have overall suggested the involvement of the fronto-striatal and mesocorticolimbic reward circuitry in these behavioral deficits in BN. The impaired self-regulatory capacity has been associated with deficient activity in the dorsolateral and inferior frontal gyrus observed when BN patients performing impulsive inhibition tasks^3–5^. The brain activation changes detected using reward tasks is inconclusive. Whereas studies reported reduced activation in the mesocorticolimbic areas during a taste-reward learning test^6^, and in the fronto-striatal circuitry during reward-based spatial learning^7^, others reported increased prefrontal and striatal activation during probabilistic category learning^8^ and food rewarding^9–11^. The body image distortion of BN has been associated with functional disturbances in the somatosensory and visuospatial networks, such as reduced occipitotemporal response during the presentations of abnormal-weight bodies^12^ and resting-state functional connectivity (RSFC) within the somatosensory and occipital cortex^13^. Reduced parietal activation was also observed when BN patients viewing their own body^14^ and processing self-referent emotional information^15^.

These studies mainly examined task-elicited regional activation, which provides a limited window into whole-brain network changes of BN patients. By analyzing the functional brain network based on resting-state fMRI (R-fMRI) data of 44 BN and 44 healthy women, we found increased nodal strength and RSFC across the primary sensorimotor cortex, parietal and occipital areas in BN patients, but reduced changes within the mesocorticolimbic reward circuitry^16^. Several studies have suggested that these functional changes have a structural basis. Using voxel-based analysis and tract-based spatial statistics, studies^17,18^ demonstrated impaired white-matter (WM) integrity in BN patients in multiple fiber tracts including the corona radiata, corpus callosum, fornix, superior and inferior fronto-occipital fasciculus, uncinate fasciculi, anterior thalamic radiation, and cingulum, which connect through the prefrontal, temporal, limbic, subcortical systems, and the regions between two hemispheres. Another study examining the taste-rewarding circuitry exhibited altered structural connections among the insula, orbitofrontal cortex (OFC), and ventral striatum in both bulimia and anorexia nervosa (AN) patients^19^. However, these studies have focused on specific WM tracts or connections within the pre-defined circuitry. They could not identify exactly which regions are interconnected by the affected tracts and the effects of abnormally connected regions on the information exchange within the whole brain, which is thus lack of a global perspective.

Connectomics provide a powerful way for comprehensive mapping of whole-brain networks. A number of topological properties, including small-worldness, network hubs, and modularity, have been characterized in the human brain^20^. The analysis of nodal properties could identify the regional connectivity of a specific node and its effect on the whole brain^20^. By segmenting the whole brain as an interconnected network, the network-based statistic (NBS) provides an unbiased means for testing network-level abnormalities affected by diseases^21^. However, there has been no study conducted to examine the nodal properties and inter-regional structural connectivity within the whole brain of BN patients.

Here, we collected the diffusion tensor imaging (DTI) data of 48 BN women and 44 healthy controls (HCs) and employed connectomic approaches (including the nodal properties and NBS) to analyze whole-brain network changes in BN patients. Based on the above-noted evidence of psychological characteristics of BN and their neural correlates revealed by functional imaging studies, the impaired WM tracts, and altered RSFC observed previously by our group, we predicted that disrupted nodal properties and connections would be evident in the PFC that serves self-regulatory control, the mesocorticolimbic circuitry concerned with reward and emotional processing, the somatosensory and visuospatial networks that support body image processing.

## Subjects and methods

### Subjects

The sample consisted of 48 BN women and 44 HC women. Those with BN were recruited from the outpatient and inpatient services of Peking University Sixth Hospital. Control subjects were recruited via local advertisements. The diagnosis of BN was made by a psychiatrist with expertise in ED using the Mini International Neuropsychiatric Interview (MINI)^22^, a short structured interview developed according to the DSM-IV criteria. Participants with current Axis I psychiatric disorders were excluded. For BN patients, a physical examination was performed and the indicators of nutritional status such as blood biochemistry and electrolytes were examined to exclude the conditions of malnutrition. Among the 48 patients, 20 have a history of antidepressant medication, but all of them have no use of psychotropic medications for at least one month prior to the study. Seventeen patients have a history of AN.

Control subjects were required to have normal body weight and menstrual cycles, and no lifetime history of any psychiatric disorders. All subjects were required to be right-handed, have no history of neurological diseases, mental retardation, unstable medical conditions, or substance use disorders within the last year. The authors asserted that all procedures contributing to this work comply with the ethical standards of the Institutional Review Board of Peking University Sixth Hospital on human experiments and with the Helsinki Declaration. Written informed consent was obtained from all subjects.

### Clinical assessment

All subjects completed the Eating Disorder Inventory-1 (EDI-1)^23^, HAMD, and HAMA. A psychiatrist who has received the consistency training conducted the assessment of HAMD and HAMA. The EDI-1 is a 64-items self-report questionnaire of psychological traits clinically relevant to ED^23^. Participants respond on a 6-point Likert scale (“Always” through to “Never”). This study reported the EDI-1 subscales of drive for thinness, bulimia, body dissatisfaction, and interceptive deficit, considering that they are more directly relevant to BN than other subscales. The latest evaluation edition for EDs is EDI-3, but since the reliability and validity study of EDI-3 and 2 have not completed in Chinese population, which is actually the things our group are doing, we used EDI-1 for evaluation in this study. The reliability and validity of EDI-1 have been verified in people with EDs in China^23^. Of note, the 1 and 3 editions of EDI have a common assessment content for the subscales examined in this study.

### MRI acquisition

Diffusion-weighted images were acquired on a Siemens 3.0 Tesla scanner at the People’s Liberation Army 306 Hospital with the following parameters: 30 diffusion directions with *b*-value 1000 s/mm^2^; repetition time/echo time (TR/TE), 6100/93 ms; acquisition matrix, 128 × 128 × 45; voxel size, 1.9 × 1.9 × 3.5 mm; field of view, 240 × 240 mm^2^; 90° flip angle. For a registration propose, T1-weighted structural images were obtained using magnetization-prepared rapidly acquired gradient-echo (MPRAGE) sequence: TR/TE, 2300 ms/3.01 ms; thickness/gap, 1.0/0 mm; matrix, 256 × 256; voxel size, 1 × 1 × 1 mm^3^; 9° flip angle. No subjects displayed obvious structural damage based on their conventional MRI scans.

### Data preprocessing

DTI preprocessing was performed using FMRIB’s Diffusion Toolbox (FSL, http://www.fmrib.ox.ac.uk/fsl/fdt/index.html). The eddy current distortions and motion artifacts were corrected by applying a rigid-body transformation of each diffusion-weighted image to the b0 image. Afterwards, each b0 image was registered to T1-weighted images, and then to MNI-152 space. The transformation matrix from diffusion to MNI space was calculated by the matrices generated in above registering steps. Fiber tracts were reconstructed in the diffusion toolkit toolbox using the Fiber Assignment by Continuous Tracking (FACT) algorithm. The starting points were chosen spatially at random within the voxel where the fractional anisotropy (FA) value was >0.3^24^. The tracking procedure was terminated at voxels with a FA value <0.15 or when the angle between adjacent steps was >45°^24^. To enable group comparisons, the diffusion images were registered to MNI-152 space using the transformation matrix.

### Network construction

Nodes and edges are two elements of the brain network. For the definition of nodes, the whole brain was divided into 90 cortical and subcortical regions of interest (45 on the left side and 45 on the right side) according to the Automated Anatomic Labeling (AAL) atlas^25^, and each region represents a node of the network. The AAL atlas is provided by the Montreal Neurological Institute (MNI). There are 116 regions in the AAL template, but only 90 regions belong to the cortical and subcortical structures, and the remaining 26 regions belong to the cerebellum. For the edge of the network, we used the fiber tracking in the FSL and build a binary network by sparse threshold method. According to previous DTI studies^26–28^ of our group, two regions were considered as anatomically connected if the fiber tracts passing through their respective nodes were at least 2 fibers with the size >5 mm. We set these threshold values for edges to reduce the risk of false-positive connections. The weighted FA values were computed as the average over all points and included stream-lines which connected two separate regions. After that, the FA-weighted network was constructed.

To test the reliability of constructed network, we computed the intra-class correlation (ICC), which has been recommended to quantify the reliability of connection matrix^29–31^. The ICC was computed using MATLAB toolbox created by Arash Salarian (www.mathworks.com/matlabcentral/fileexchange/22099). The ICC values are categorized into 5 common intervals^31^: 0 < ICC ≤ 0.2 (slight); 0.2 < ICC ≤ 0.4 (fair); 0.4 < ICC ≤ 0.6 (moderate); 0.6 < ICC ≤ 0.8 (substantial); and 0.8 < ICC < 1.0 (almost perfect). It is considered that the larger the ICC value, the better the reliability of connection matrix^29,30^. A network with moderate-to-almost perfect test-retest reliability (ICC ≥ 0.4) is commonly expected in practice^29^.

### Nodal characteristics

The four indices of nodal properties including nodal strength *S*, global efficiency *E*_glob_, local efficiency *E*_loc_, and betweenness centrality *B*, were calculated using the brain connectivity toolbox for normalized weighted structural networks. For each subject, we chose the nodes that exhibited significant group differences in at least one of the four nodal properties. The definition of the nodal metrics and calculating methods were provided in the supplemental material. For the nodal indices, the nonparametric permutation test was used to examine the differences between BN and HC groups, with the *p* value corrected by false discovery rate (FDR). To examine the possible effect of AN history on the results, we compared the nodal indices between BN patients with and without AN history.

### NBS

We used the NBS approach to localize the regional pairs in which the WM connections were altered in BN patients. The NBS is a statistical tool to deal with the multiple comparisons problem when analyzing graphs^21^. We first identified an undirected graph of 90 nodes for each subject. For each pairwise association, the test statistic of interest was calculated independently using the values stored in each subject’s connection matrix. A method was used to detect the non-zero connections. For the weighted FA network of BN an HC group, if the FA value of more than half of the subjects are zero in a connection, the connection is set zero. A nonparametric one-tailed sign test in each group was added to detect the significant non-zero connections, which was corrected by FDR. Then, a two-sample *t*-test was used to compare the connection matrices between patients and controls, with 5000 permutations and setting the significant *p*-value at 0.05 corrected for multiple comparisons. Permutation testing was used to ascribe a *p*-value controlled for the FWE to each connected component based on its size. For each permutation, the test statistic of interest was recalculated, after which the same threshold was applied to define a set of supra-threshold links. To facilitate data interpretation, we sorted connections based on the functional hierarchy^32^, which divided the whole brain into the primary sensorimotor, unimodal, heteromodal, paralimbic, limbic, and subcortical systems.

### Correlations with clinical variables

We performed the correlation analysis between nodal properties showing significant group differences and clinical variables (including illness duration, EDI subscales (i.e., body dissatisfaction, bulimia, drive for thinness, and interceptive awareness)) within BN patients, while controlling age, educational level, and current BMI. We additionally examined correlations between the nodal measures and scores of HAMD and HAMA to identify the possible effects of depressive and anxiety symptoms. Alpha level was not corrected due to the exploratory nature of this study. The same correlation analysis was also performed in HCs. We did not examine the correlation between the inter-regional connectivity changes and clinical variables, given that the connections obtained by NBS is sparse, it is possible that only a part of the same connections among different subjects have a value, and others are zero, making it unfeasible to perform a correlation analysis.

## Results

### Demographic and clinical data

As shown in Table 1, there were no significant differences in age, years of education, or current BMI between BN patients and HCs; however, BN patients were significantly higher on the EDI, HAMD, and HAMA than HCs.

**Table 1:** Demographic and clinical data

Variables	Bulimia Nervosa (*n* = 48)	Healthy Control (*n* = 44)	Statistics	
			*t*	*p*
Age (years)	22.0 ± 3.4	23.1 ± 3.4	−1.507	0.136
Education (years)	14.1 ± 1.9	14.6 ± 1.7	−1.315	0.192
Current BMI (kg/m^2^)	21.0 ± 2.6	20.5 ± 1.4	1.125	0.264
Lowest previous BMI (kg/m^2^)	17.9 ± 2.3			
Highest previous BMI (kg/m^2^)	25.2 ± 4.3			
Duration of illness (years)	2.0 ± 1.3			
Drive for thinness	35.2 ± 7.2	19.4 ± 6.3	7.388	<0.001
Bulimia	34.0 ± 5.8	13.0 ± 5.0	12.086	<0.001
Body dissatisfaction	41.9 ± 8.8	27.1 ± 7.1	5.725	<0.001
Interoceptive awareness	38.4 ± 8.1	20.9 ± 6.5	7.418	<0.001
17-HAMD	6.7 ± 3.3	0.9 ± 1.0	11.013	<0.001
HAMA	5.1 ± 3.3	0.4 ± 0.7	9.405	<0.001

*Note*: data are presented as mean ± SD

*BMI* body mass index, *HAMD* Hamilton Rating Scale for Depression, *HAMA* Hamilton Anxiety Rating Scale

### Nodal characteristics

**Fig. 1:**
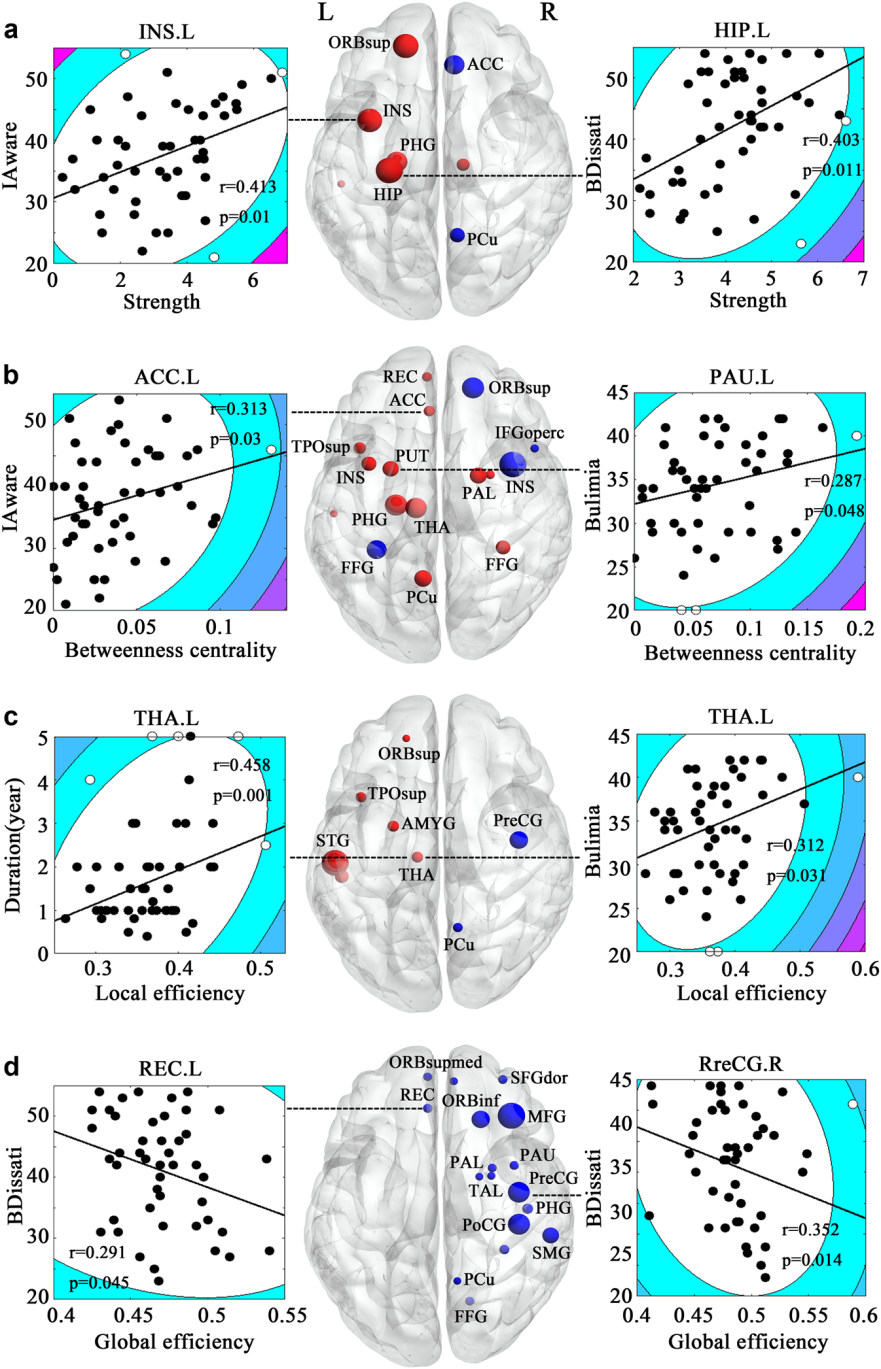
Group differences in nodal properties. **a** Nodal strength; **b** betweenness centrality; **c** local efficiency; **d** global efficiency. The red colors indicate the nodes showing higher degree in bulimia nervosa patients compared to healthy controls, while the blue colors indicate the opposite. The size of the spheres indicates the statistical significance of nodal degree changes. ORBsub, superior frontal gyrus, orbital part; REC gyrus rectus, SFGdor superior frontal gyrus, dorsolateral, MFG middle frontal gyrus, PreCG precentral gyrus, PoCG postcentral gyrus, ACC anterior cingulate cortex, STG superior temporal gyrus, HES heschl gyrus, PCu precuneus, SMG supramarginal gyrus, PHG parahippocampal gyrus, HIP hippocampus, FFG fusiform gyrus, LG lingual gyrus, INS insula, PUT putamen, PAL pallidum, THA thalamus, IAware interceptive awareness, BDissati body dissatisfaction. Significance of correlations between nodal properties and clinical variables are not corrected for multiple comparisons

Compared with HCs, BN patients showed increased nodal strength changes in the left superior OFC, left inferior temporal gyrus (ITG), left insula, left hippocampus, left parahippocampal gyrus (PHG), left thalamus but reduced changes in the left anterior cingulate cortex (ACC) and right precuneus (Fig. 1a). We observed increased betweenness changes in the left superior medial OFC, left ACC, left STG, left superior temporal pole, left Precuneus, right fusiform gyrus, left insula, left PHG, left putamen, right pallidum, left thalamus, right amygdala but reduced changes in the right IFG, right superior OFC, left fusiform gyrus, and right insula (Fig. 1b). For the local efficiency, we observed increased changes in the left superior OFC, left STG, left ITG, left superior temporal pole, left thalamus, left amygdala but reduced changes in the right precentral gyrus (PreCG) and right precuneus (Fig. 1c).

Conversely, multiple nodes in the dorsolateral PFC, mesocorticolimbic reward circuitry (OFC, gyrus rectus, insula, and subcortical areas (putamen, pallidum, amygdala)), somatosensory and visuospatial networks (PreCG, postcentral gyrus (PoCG)), parietal (supramarginal gyrus (SMG), PCu)) and occipital (FFG) cortex showed reduced global efficiency, with most of them distributed over the right hemisphere (Fig. 1d). The left gyrus rectus and right PreCG were negatively correlated with the EDI subscale of body dissatisfaction. See Fig. 1 and Table 2 for more details. No significant correlations were observed between the nodal measures and scores of HAMD and HAMA. And also, no significant correlations were observed between the nodal measures and clinical variables within HCs. We have not found any significant differences between the BN patients with and without AN history. So, it could be concluded that the AN history has no impact on the current results.

**Table 2:** Between-group differences in nodal properties

	*p* value, properties (BN-HC)/NC			
Regions	Strength	Betweenness	Local efficiency	Global efficiency
SFGdor.R				0.0054, −43.53%
MFG.R				0.00004, −24.11%
IFGoperc.R		0.0009, −26.37%		
ORBsup.L	0.0027, 22.43%		0.0023, 15.43%	
ORBsup.R		0.00002, −17.20%		
ORBmid.L				
ORBmid.R				0.0011, −25.03%
ORBsupmed.L		0.0005, 37.90%		0.0025, −38.93%
ORBsupmed.R				0.0086, −28.30%
ORBinf.R				0.00004, −31.91%
Gyrus rectus.L				0.0034, −21.23%
PreCG.R			0.00002, −38.79%	0.0002, −29.35%
PoCG.R				0.0013, −23.49%
SMG.R				0.0105, −39.62%
ACC.L	0.0008, −22.49%	0.0021, 38.54%		
STG.L		0.0019, 27.41%	0.00002, 36.17%	
ITG.L	0.0014, 52.17%		0.00002, 42.59%	
TPOsup.L		0.0001, 23.36%	0.00002, 33.36%	
HES.L				
Precuneus.L		0.0001, 39.93%		
Precuneus.R	0.0021,−23.23%		0.0004, −15.6%	0.0081, −19.18%
FFG.L		0.00002, −20.38%		
FFG.R		0.00004, 14.01%		0.0003, −26.72%
Insula.L	0.002, 47.07%	0.0005, 24.97%		
Insula.R		0.0001, −32.82%		0.0018, −22.16%
Hippocampus.L	0.0004, 37.47%			
PHG.L	0.0001, 28.11%	0.0006, 39.93%		
PHG.R				
Putamen.L		0.0009, 36.56%		
Putamen.R				0.0041, −20.44%
Pallidum.L				
Pallidum.R		0.0005, 18.73%		0.0091, −32.14%
Caudate.L				
Thalamus.L	0.0023, 23.61%	0.0008, 43.91%	0.0002, 18.73%	
Thalamus.R				
Amygdala.L			0.00002, 31.35%	
Amygdala.R		0.0013, 47.26%		0.0022, −23.58%

*SFGdor* superior frontal gyrus, dorsolateral, *MFG* middle frontal gyrus, *IFGoperc* inferior frontal gyrus, opercular part, *ORBsup* superior frontal gyrus, orbital part, *ORBmid* middle frontal gyrus, orbital part, *ORBsupmed* superior frontal gyrus, medial orbital, *ORBinf* inferior frontal gyrus, orbital part, *PreCG* precental gyrus, *PoC*G postcentral gyrus, *SMG* supramarginal gyrus, *ACC* anterior cingulate cortex, *STG* superior temporal gyrus, *ITG* inferior temporal gyrus, *TPOsup* temporal pole: superior temporal gyrus, *HES* heschl gyrus, *FFG* fusiform gyrus, *PHG* parahippocampal gyrus, *L* left, *R* right

### NBS

The ICC value of network metrics across all BN subjects is 0.9388, and the ICC value of network metrics across all HC subjects is 0.9977, which is almost perfect, suggesting a very good reliability of the connection matrix.

**Fig. 2:**
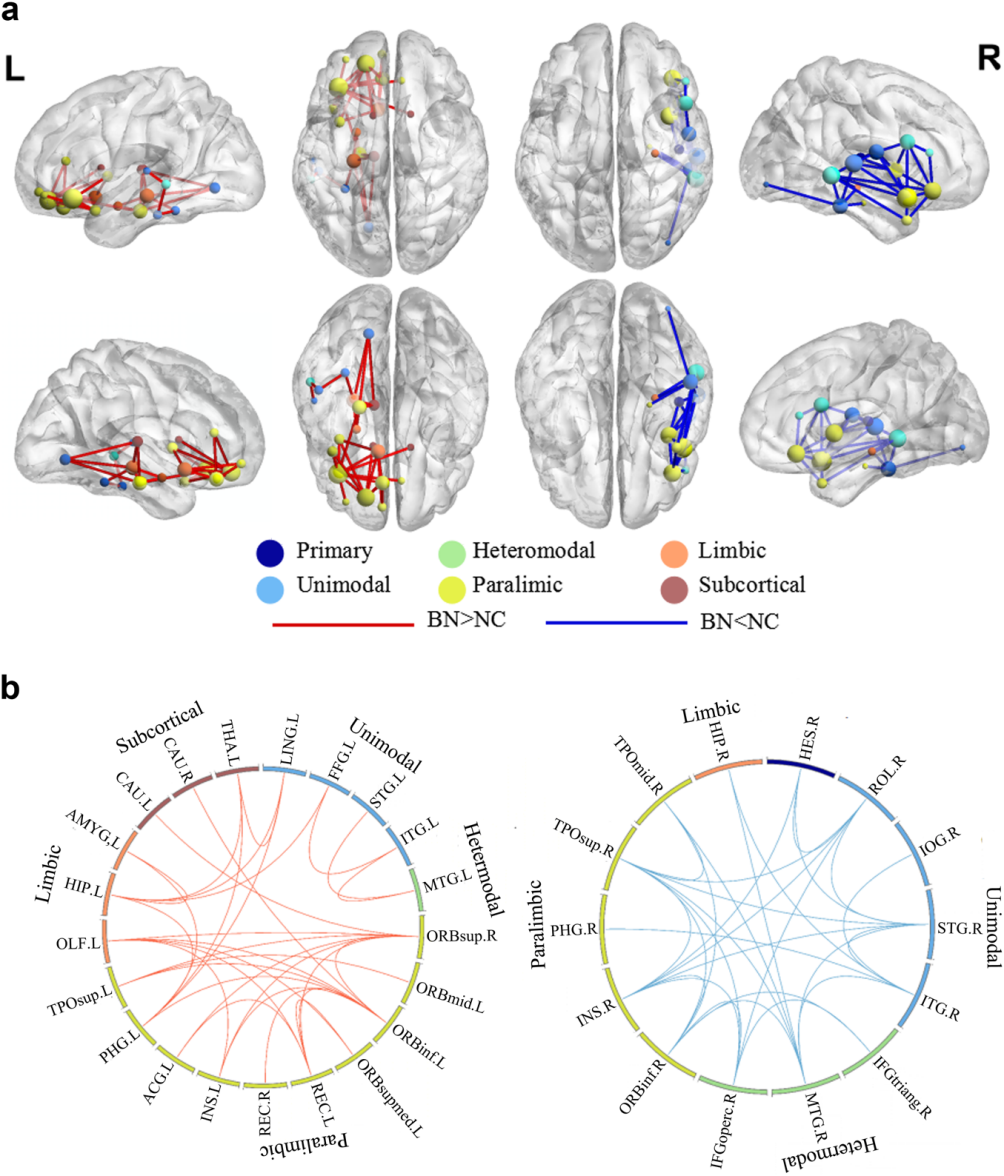
Group differences in structural connections obtained by network-based statistics. **a** Both higher (red lines) and lower (blue lines) connections in bulimia nervosa (BN) patients compared to healthy controls (HCs). The thickness of red lines indicates the number of edges showing higher connections in BN patients compared to the HCs, while the thickness of blue lines indicates the opposite. The spheres were marked in different colors according to their functional divisions. **b** The circle maps show specific brain regions with altered connections. ORBsub superior frontal gyrus, orbital part, ORBmid middle frontal gyrus, orbital part, ORBinf inferior frontal gyrus, orbital part, IFGtriang inferior frontal gyrus, triangular part, IFGoperc inferior frontal gyrus, opercular part, SFGdor superior frontal gyrus, dorsolateral, MFG middle frontal gyrus, REC gyrus rectus, PreCG precentral gyrus, PoCG postcentral gyrus, ACC anterior cingulate cortex, STG superior temporal gyrus, MTG middle temporal gyrus, ITG inferior temporal gyrus, TPOsup temporal pole: superior temporal gyrus, TPOmid temporal pole: middle temporal gyrus, HES heschl gyrus, SMG supramarginal gyrus, PCu precuneus, FFG fusiform gyrus, IOGinferior occipital gyrus, LG lingual gyrus, ROL rolandic operculum, OLF olfactory cortex, INS insula, PHG parahippocampal gyrus, HIP hippocampus, CAU caudate, PUT putamen, PAL pallidum, THA thalamus, AMYG amygdala, L left, R right

The between-group comparisons showed altered connections in a total of 65 edges in BN patients compared to the HCs, with 36 increased and 29 decreased. Sorted by the functional hierarchy^32^, we found increased connections within the mesocorticolimbic reward circuitry involving the paralimbic, limbic, and subcortical areas. It could be seen from Fig. 2 that increased connections involved multiple edges within the OFC, between the OFC and paralimbic/subcortical areas including the ACC, insula, caudate, and thalamus. The increased connections also included several edges within and between the lateral temporal-occipital cortex and paralimbic system (OFC, PHG). Conversely, reduced connections primarily involved the edges within the paralimbic areas (IFG, insula) and their connections to lateral temporal cortex. Interestingly, the increased connections were distributed over the left hemisphere, while decreased connections presented a right-lateralized dominance (Fig. 2, Table S1).

## Discussion

This study is to our knowledge the first to examine BN-related changes in whole-brain WM network using the DTI technology. The primary results included the 3 aspects: (1) Increased nodal properties in multiple left-lateralized areas within the mesocorticolimbic reward circuitry, lateral temporal-occipital cortex, and PCu, but reduced global efficiency in right-lateralized areas within the dorsolateral PFC, mesocorticolimbic circuitry, somatosensory and visuospatial networks in BN patients compared to HCs. (2) Significant correlations between nodal properties of the mesocorticolimbic reward areas and the EDI subscales. (3) Increased connections primarily within the mesocorticolimbic reward circuitry involving the paralimbic, limic, and subcortical areas, between the OFC and temporal-occipital cortex, but reduced connections across the IFG and lateral temporal cortex. Interestingly, the nodal and connection changes presented a hemispheric-specific change with left increase but right decrease.

The first important finding is the left-lateralized increases in nodal properties and connections among the OFC and other mesocorticolimbic reward areas. The OFC, in conjunction with the paralimbic/limbic and subcortical areas, constitute the mesocorticolimbic circuitry important for reward and emotional processing^33,34^. Substantial evidence has suggested the involvement of the mesocorticolimbic circuitry in taste-rewarding disturbances of binge eating groups. For instance, greater gray-matter volume was observed in the medial OFC of BN patients, which predicted taste pleasantness^35^. Whereas a fMRI study exhibited reduced activation in multiple mesocorticolimbic areas when BN patients performing taste-reward learning test^6^, more studies reported increased mesocorticolimbic response as detected during probabilistic categories learning^8^, reward anticipation^36^, and receipt of food stimuli^9–11^. Directly related to our study is the impaired WM integrity in BN patients in the uncinate fasciculi, anterior thalamic radiation, and cingulum tracts^17,18^ connecting the paralimbic, limbic, and subcortical areas, and increased connections in WM pathways connecting the OFC, insula, and ventral striatum^19^. The insula might be associated with EDs by playing a central role in interoceptive awareness and in emotional evaluation to external stimuli^37^. Researchers have observed an exaggerated insular response to taste stimuli in BN patients^38,39^. The significant correlations between the nodal properties in the putamen and thalamus and bulimia symptoms, between the nodal properties in the insula and ACC and interceptive awareness might indicate the behavioral implication of our results more specifically. It was shown that higher properties in the putamen and thalamus, more severe bulimia symptoms, whereas higher properties in the insula and ACC, more severe deficits of interceptive awareness. Combining the physiological significance of these brain regions and their results in previous imaging studies of EDs^6,8,36–39^, we speculate that strengthened nodal degree in the putamen and thalamus might contribute to excessive appetitive responsivity and taste-sensory transmission, which is driven by abnormally high emotional significance attribute to taste stimuli mediated by strengthened nodal degree in the insula and ACC. These disturbed neural processes collectively contribute to the binge eating behaviors.

The nodal and connectivity increases in left-lateralized mesocorticolimbic circuitry seems to be not compatible with a previous R-fMRI study of our group^16^, which used a BN and HC sample included in this study and exhibited a functional change in BN patients contrary to this structural investigation. However, the results of that study and this work are not contradictory, given that despite the left-lateralized increases, the right-lateralized decreases in global efficiency were found in the mesocorticolimbic reward regions, suggesting a weakened effect of these areas on the informational communication within the whole brain. Therefore, both the structural and functional changes might indicate an inefficiency of the mesocorticolimbic and fronto-striatal circuitry in BN.

Contrary to the increased changes in the OFC, we observed decreased nodal properties and connections in the right-lateralized IFG and dorsolateral PFC of BN patients. The IFG and dorsolateral PFC support response inhibition or conflict resolution^40^, deficits of which were pervasively existed in binge eating persons^3,4^. Accordingly, BN patients have been failed to activate the dorsolateral PFC and IFG when engaging self-regulatory processes necessary to resolve conflicting^3^, which occurred even in less severe clinical symptoms^4,7^. Other supporting evidence includes the findings of impaired prefrontal control over interference by food images in binge eating patients^10^ and a high predictive potential of the IFG volume for bulimia symptoms and Stroop interference^41^. It is therefore possible that the weakened connections to the IFG and dorsolateral PFC lay a structural foundation for the self-regulatory and related neural deficits in BN. These functional and structural disturbances release feeding behaviors from regulatory control, thereby perpetuating the conflicting desires to binge eating.

The hemispheric-specific change in the prefrontal and mesocorticolimbic reward areas is an interesting phenomenon. Decades of researches have linked higher approach motivation to greater left prefrontal asymmetry^42^, whereas lower behavioral inhibition sensitivity to greater right prefrontal asymmetry^43^. The prefrontal asymmetry has been associated with disinhibition and appetitive responsivity among the binge eating persons. As evidenced, a single photon emission computed tomography (SPECT) study^44^ showed that exposure to food could elicited a greater response of regional cerebral blood flow in the left-, relative to right-sided PFC in binge eating than non-binge eating women, suggesting an important role of the left hemisphere and its prefrontal regions in binge eating behaviors. Two electroencephalogram (EEG) studies showed that the prefrontal asymmetry moderated the association between the attentional bias toward food and BMI ^45^, and disinhibition, hunger, and appetitive responsivity predicted left- greater than right-sided PFC activation independent of affect^46^. Moreover, the R-fMRI studies exhibited impaired inter-hemispheric functional incoordinations in the dorsolateral PFC and OFC of BN patients^47,48^. Therefore, the lateralized changes in WM pathways through the prefrontal and mesocorticolimbic areas might be a structural basis for taste-rewarding and impulsive control disturbances of BN. More specifically, the left-lateralized increases across the OFC and mesocorticolimbic structural connections might contribute to a high behavioral activation or reward sensitivity to taste stimuli, while the right-lateralized connection reduction in the IFG likely underlie impaired cognitive control over binge eating and other impulsive behaviors.

The results important for understanding body image distortion is the structural changes in somatosensory and visuospatial networks of BN patients. Specifically, the nodal properties were altered in the primary sensorimotor cortex (PreCG, PoCG), lateral temporal cortex, parietal (SMG, PCu) and occipital (FFG) cortex. The structural connections were increased in the left-sided temporal-occipital cortex but reduced in more extensive right-sided temporal areas. The perception and integration of body image information depend on a large network involving the primary sensorimotor cortex, the temporal, parietal, and occipital visual association cortex^32,49^. Researchers observed reduced occipitotemporal response during the presentations of abnormal weight bodies^12^ and reduced RSFC across the somatosensory network and occipital cortex^13^, and our group found increased nodal strength and RSFC in the primary sensorimotor cortex and occipital areas^16^ in BN patients. The DTI studies exhibited impaired WM integrity in patients with BN^18^ and body dysmorphic disorder^50^ in the superior longitudinal, inferior fronto-occipital, and uncinate fasciculi that connect the prefrontal, temporal, and occipital visual association cortices; FA reductions in these tracts were greatest in patients who were most preoccupied with their body shape^18^. The parietal areas (including the SMG and PCu) are worth additionally emphasized given their central roles in self-related cognitive activity^51^, which therefore might be associated with the characteristics of undue influence of body shape and weight on self-evaluation of ED patients^52^. Together, these structural changes in the somatosensory and visuospatial networks might be fundamental to the functional disturbances of inappropriate sensory and visual processing of body image in BN patients.

Intriguingly, the lateral temporal connections also showed a phenomenon of hemispheric-specific change, with a left-increased but right-decreased change and altered structural connections on the right more than left hemisphere. This finding is compatible with the right-lateralized dominance for body schema^53^. In support of our current study, researchers observed reduced WM volume in the right-sided temporal cortex in both the AN and BN groups^35^ and impaired WM integrity in the corpus callosum of BN patients^17^, which connects left and right hemispheres and transmits the information from the brain regions between two hemispheres. The evidence raises the possibility that the hemisphere-specific change in the lateral temporal cortex might be another neural mechanism underlying distorted body imaging of BN.

Several factors distinguish our study from past investigations. This is the largest sample study of BN using DTI technology so far. The recruitment of unmedicated and acute patients with short illness duration has largely disentangled the effects of medication and chronic course. We excluded the patients with moderate to severe depressive symptoms and performed the correlation analysis between depressive and anxiety severity and nodal measures, with no significant correlations found. We could therefore largely exclude the confounding effects of depression and anxiety on the WM structural changes of BN.

However, several issues need to be further addressed. First, the precise regional pairs linked by disrupted connections should be interpreted cautiously due to the possibility for diffusion tractography to generate false positives and/or negatives in tract reconstruction, which is a common limitation to DTI studies. The solution to this problem will benefit from the development of novel acquisition sequences and tractography algorithms. Second, we noted that BN-related impairments in WM integrity has been observed in a teens group^18^, a critical period that most BN patients starts. Future researches comparing the structural networks between adult and adolescent patient groups will help to determine development-related changes. Third, the disorder specificity of the findings to BN remains to be clarified given that studies^54–56^ investigated the WM integrity in mood, psychotic, and substance use disorders found reduced FA in a multitude of regions overlapped with our study. A direct comparison with AN and other patient groups will be necessary to clarify this issue. Finally, we explained the results of hemisphere-specific change by combining several fMRI and EEG studies, but the underlying neural mechanisms need to be clarified by more specific experimental designs. For example, to use the transcranial magnetic stimulation (TMS)-EEG technology to perturb the left and right brains of BN patients might be a proper design to examine the differences in neural response between two hemispheres. Further research combining a resting and task design to specify the precise role of asymmetry in the motivation toward feeding, self-regulation, and body imaging in BN individuals is encouraged.

In conclusion, this study revealed nodal and connectivity changes distributed over the PFC, the mesocorticolimbic reward circuitry, the somatosensory and visuospatial networks in BN. The hemisphere-specific change could be an important aspect of the pathophysiology of BN. By characterizing the WM networks in a whole-brain view, our study provides novel insights into the structural basis of behavioral and functional deficits of BN. Future researches that integrate brain structure, function, and more specific behavioral tests would help to clarify how the WM structure affects behaviors of BN patients.

## Supplementary information

Supplemental information
