# Supplementary material for: Abnormal structural brain network and hemisphere-specific changes in bulimia nervosa
Source: Transl Psychiatry. 2019 Aug 27;9:206. doi: 10.1038/s41398-019-0543-1 (PMC6712015; doi:10.1038/s41398-019-0543-1)
Supplement: Supplementary file 1 — Supplemental information [file 41398_2019_543_MOESM1_ESM.docx]

**Supplemental information of “[Abnormal structural brain network and hemisphere-specific changes in bulimia nervosa](https://www.ncbi.nlm.nih.gov/pubmed/29755374)”**

**Methods**

**The computation of nodal characteristics**

The overall nodal characteristics including nodal strength S, nodal global efficiency E_glob_, nodal local efficiency E_loc_, and nodal betweenness centrality B, were calculated using the brain connectivity toolbox for normalized weighted brain structural networks.

**Nodal strength**

The nodal strength S was defined as the sum of the weights of direct connections in one node. The S_i_ for node i was defined as the sum of the weights of direct connections of node i:

$S_{i}=\sum_{j\in N} W_{ij}$ (1)

Where N was the set of all nodes in the graph, and W_ij_ was the weight between node i and node j in the graph. Where t_i_ referred to the actual number of edges in G_i_ (the subgraph of neighbors of i) and k_i_ meant the number of nodes in G_i_.

**Betweenness centrality**

The nodal betweenness centrality B was calculated as the fraction of shortest paths that passed through the node between other nodes, represented a central node that played pivotal role over the information transfer within the graph. Formally:

$B_{i}=\frac{1}{(n-1)(n-2)}\sum_{h,j\in N,h\neq i,j,i\neq j} \frac{sp_{hj}(i)}{sp_{hj}}$ (2)

where $sp_{hj}(i)$ was the number of shortest path between node h and node j passing through node i, and N was the set of all nodes in the graph.

**Local efficiency**

The nodal local efficiency E_loc_ was the global efficiency that computed on the neighboring nodes, measured the extent of information transmission among the adjacent nodes.

$E_{loc_{i}}=\frac{1}{N}\sum_{i\in G} E_{glob_{i}}$ (3)

**Gobal efficiency**

The nodal global efficiency E_glob_ reflects the extent of information transmission of the node with all other nodes in the network, was computed as the average of the inverse of the distances of all nodes that directly connected with the node except the node itself. Formally:

$E_{glob_{i}}=\frac{1}{N-1}\sum_{j\in N,j\neq i} \frac{1}{d_{ij}}$ (4)

Where d_ij_ was the shortest path length between node i and j in G, and N was the set of all nodes in the graph.

**Table 1.** **Between-group differences in structural connections obtained by network-based statistics.**

| **Bulimia nervosa > Healthy control** | **Bulimia nervosa < Healthy control** |
| --- | --- |
| **Within the paralimbic system** | **Within the paralimbic system** |
| ORBsup.L-ORBinf.L | IFGoperc.R-IFGtriang.R |
| ORBsup.L-Gyrus rectus.L | IFGoperc.R-ORBinf.R |
| ORBinf.L-Gyrus rectus.L | IFGtriang.R-ORBinf.R |
| ORBsupmed.L-Gyrus rectus.L | IFGoperc.R-ROL.R |
| ORBinf.L-Gyrus rectus.L | IFGoperc.R-Insula.R |
| ORBsup.L-Insula.L | ORBinf.R-Insula.R |
| ORBinf.L-Insula, left | ROL.R-Insula.R |
| ORBsup.L-ACC.L | **Between paralimbic and unimodal/hetermodal systems** |
| Gyrus rectus.L-ACC.L | IFGoperc.R-STG.R |
| ORBinf.L-PHG.L | ORBinf.R-STG.R |
| **Between paralimbic and subcortical systems** | ORBinf.R-MTG.R |
| ORBsup.L-Caudate.L | ROL.R-STG.R |
| ORBinf.L-Caudate.R | PHG.R-ITG.R |
| PHG.L-Thalamus.L | Insula.R-STG.R |
| ORBsup.L-Caudate.L | Insula.R-STG.R |
| ORBinf.L-Caudate.R | ORBinf.R-MTG.R |
| PHG.L-Thalamus.L | **Within unimodal/hetermodal systems** |
| **Between paralimbic and limic systems** | ROL.R-STG.R |
| ORBsup.L-OLF.L | STG.R-MTG.R |
| ORBinf.L-OLF.L | STG.R-MTG.R |
| ORBsupmed.L-OLF.L | STG.R-MTG.R |
| Gyrus rectus.L-OLF.L | MTG.R-MTG.R |
| Insula.L-OLF.L | IOG.R-ITG.R |
| PHG.L-Hippocampus.L | STG.R-STG.R |
| PHG.L-Amygdala.L | STG.R-ITG.R |
| **Between imbic and subcortical systems** | MTG.R-ITG.R |
| Hippocampus.L-Thalamus.L | **Between limbic and unimodal/hetermodal systems** |
| Hippocampus.L-Thalamus.L | Hippocampus.R-ITG.R |
| **Within the limbic system** | Hippocampus.R-MTG.R |
| Hippocampus.L-Amygdala.L | **Between primary and paralimbic systems** |
| **Between paralimbic/limbic and unnimodal systems** | HES.R-Insula.R |
| ORBsup.L-STG.L | **Between primary and unimodal systems** |
| ORBmid.L-STG.L | ROL.R-HES.R |
| ORBinf.L-STG.L | HES.R-STG.R |
| PHG.L-LG.L |  |
| Hippocampus.L-LG.L |  |
| Hippocampus.L-FFG.L |  |
| **Between unnimodal and hetermodal systems** |  |
| STG.L-MTG.L |  |
| FFG.L-ITG.L |  |
| MTG.L-ITG.L |  |
| **Between unimodal and subcortical systems** |  |
| LG.L-Thalamus.L |  |

Note: ORBsub, superior frontal gyrus, orbital part; ORBmid, middle frontal gyrus, orbital part; ORBinf, inferior frontal gyrus, orbital part; IFGtriang, inferior frontal gyrus, triangular part; IFGoperc, inferior frontal gyrus, opercular part; SFGdor, superior frontal gyrus, dorsolateral; MFG, middle frontal gyrus; PreCG, precentral gyrus; PoCG, postcentral gyrus; ACC, anterior cingulate cortex; STG, superior temporal gyrus; MTG, middle temporal gyrus; ITG, inferior temporal gyrus; TPOsup, temporal pole: superior temporal gyrus; TPOmid, temporal pole: middle temporal gyrus; HES, heschl gyrus; PHG, parahippocampal gyrus; SMG, supramarginal gyrus; FFG, fusiform gyrus; IOG, inferior occipital gyrus; LG, lingual gyrus; ROL, rolandic operculum; OLF, olfactory cortex. L, Left; R, Right.
